# Poly(lactic-*co*-glycolic) acid drug delivery systems through transdermal pathway: an overview

**DOI:** 10.1007/s40204-017-0063-0

**Published:** 2017-02-06

**Authors:** Lucas Naves, Chetna Dhand, Luis Almeida, Lakshminarayanan Rajamani, Seeram Ramakrishna, Graça Soares

**Affiliations:** 1 Center for Textile Science and Technology, University of Minho, Guimarães, Portugal; 2 CAPES Foundation, Ministry of Education of Brazil, Brasília, Brazil; 3 Department of Mechanical Engineering, Center for Nanofibers and Nanotechnology, National University of Singapore, Singapore, 117581 Singapore; 4 Anti-Infectives Research Group, Singapore Eye Research Institute, Singapore, 169856 Singapore; 5 Guangdong-Hongkong-Macau Institute of CNS Regeneration (GHMICR), Jinan University, Guangzhou, 510632 China

**Keywords:** Drug delivery system, Transdermal drug delivery, PLGA, Microneedles, Electrospinning technique

## Abstract

In past few decades, scientists have made tremendous advancement in the field of drug delivery systems (DDS), through transdermal pathway, as the skin represents a ready and large surface area for delivering drugs. Efforts are in progress to design efficient transdermal DDS that support sustained drug release at the targeted area for longer duration in the recommended therapeutic window without producing side-effects. Poly(lactic-*co*-glycolic acid) (PLGA) is one of the most promising Food and Drug Administration approved synthetic polymers in designing versatile drug delivery carriers for different drug administration routes, including transdermal drug delivery. The present review provides a brief introduction over the transdermal drug delivery and PLGA as a material in context to its role in designing drug delivery vehicles. Attempts are made to compile literatures over PLGA-based drug delivery vehicles, including microneedles, nanoparticles, and nanofibers and their role in transdermal drug delivery of different therapeutic agents. Different nanostructure evaluation techniques with their working principles are briefly explained.

## Introduction

Recent advancements in the field of nanotechnology have enabled the development of new approaches for the treatment of several diseases, to minimize side-effects and to design more efficient and controlled drug delivery systems. Nanotechnology refers to the characterization, fabrication, and applications of active substances in nanometer scale dimension for various end uses (Sridhar et al. 2015). The desired drug dosage and the therapeutic window is one of the primary criteria to be considered while designing drug delivery systems (DDS). By employing nanomaterials as a base for drug delivery nanocarriers, it is now possible to encapsulate a variety of important therapeutic agents, such as nucleic acids, peptide protein-based drugs, and small molecules either hydrophobic or hydrophilic, which helps enhancing the therapeutic bioavailability at the targeted area while minimizing the toxicity in healthy cells. The stability and solubility of drugs can also be monitored by encapsulating different molecules and chemicals into the nanocarrier (Langer 1998). A preferable biological nanomaterial should possess certain characteristics, such as: chemical compatibility with physiological solutions; non-toxic, biodegradable, and biocompatible; easy to design and modify; preferably the usage of natural/biological materials (Korrapati et al. 2015).

The stratum corneum layer of the epidermis is responsible for many functions in the skin, including its role in regulating the transport of different chemical compounds into the skin. Thus, skin is a largest integumentary organ that can be approached to deliver different drugs or active compounds by topical or transdermal route. Active and passive skin penetrations have been achieved over the last few decades, improving the efficiency of either transdermal delivery (the drugs are delivered into subcutaneous tissue, therefore, taken up systemically into the body) or topical delivery (this method allows the drug delivery into skin strata). Topical therapies are an alternative treatment for several types of skin disorder as skin cancer, contact dermatitis, and psoriasis, which the drugs are delivered directly into skin strata (Zhang et al. 2014). Transdermal and topical delivery systems ensure additional advantages over other delivery routes, viz.: provide enhanced biocompatibility by evading the hepatic first-pass metabolism, support patient compliance by decreasing the drug dosage and at the same time maintaining the therapeutic effect of the drug, and enhance the bioavailability of the therapeutic agent at the targeted tissue or cells. While adopting transdermal drug delivery administration route, drug penetration is the point of prime concern. In this context, usage of nanoparticles is reported to enhance the drug penetration efficiency across the skin barrier and mucous membrane (Zheng et al. 2012).

Transdermal drug delivery system is an important route to deliver drugs into the body; the delivery through this approach is focusing in many alternatives to overcome some crucial problems related to the protective barrier of the skin. This approach may offer several advantages; since the skin is the biggest organ in the human body, it represents a relatively and readily accessible surface area for drugs absorption. The delivery of medication through transdermal route is less invasive when compared to other approaches, such as intravenous and oral route; this last approach can lead to drug degradation under extreme acidity of the stomach, and might interact with food, causing erratic delivery. The transdermal route also offers a non-invasive procedure that allows continuous intervention and monitoring. In addition to that, this approach allows ceasing the drugs or compounds absorption, preventing undesired effects and overdose (Contreras 2007). On the other hand, this method has some disadvantages, as not all compounds available worldwide are suitable as nanocarriers across the skin. The penetration rate can vary from one skin type to the other, depending upon the application site, race, age, and the type of skin disease under treatment, etc. An excellent transdermal delivery system should provide adequate release drug formulation, and, at the same time, allows considerable amount to overcome the skin barrier. It is necessary to develop biocompatible drugs to avoid skin irritation, which worsens patient’s health condition. Transdermal drug delivery must ensure that the drug will not be inactivated on the skin’s surface or even during the penetration process (Langer 2004). While developing nanofibrous wound dressings for tissue engineering, some researchers have reported enhanced proliferation of human dermal fibroblast (HDF) on electrospun mats surface (Jin et al. 2013).

In the last years, controlled-release dosage form as biocompatible and injectable biodegradable polymeric particles, including poly(lactic-*co*-glycolic acid) (PLGA), has been employed to avoid the invasive approach of surgical intervention and implants. The crystallinity of the PLGA polymer is directly related to its swelling behavior, mechanical strength, subsequently its biodegradation rate, its hydrolysis capability which further depends on the molecular weight, and the molar ratio of the lactic and glycolic in the polymer chain (Wu 1995; Gilding and Reed 1979; Lewis 1990). PLGA is approved as a material to design DDS for parental approach by the European Medicine Agency and the US Food and Drugs Administration. PLGA is used worldwide for the preparation of intravenous (DDS) and biomimetic materials, and it has extensive applications prospects in tissue engineering, medical imaging, drugs delivery, and disease diagnosis (Kocbek et al. 2007). It possesses attractive properties as drug delivery carrier, including biocompatibility and biodegradability, it protects drugs from degradation, the particles can target some specific cells, its system adapts to various types of drugs, hydrophilic or hydrophobic macromolecules, or small molecules, it has sustained release possibility, and possesses flexible surface properties that can be tuned as per the concerned application. Zhang et al. (2013) have reported the surface modification of PLGA to enhance a better efficacy of the drug, increasing the drug availability at specific areas with delivery in a sustained manner. The surface modification can be achieved by depositing few atomic layer thick biocompatible polymers coating, e.g., polyethylene glycol (PEG), to tune the hydrophilicity, stability, and the aggregation ability of the PLGA-based nanocarriers. In addition, the drug delivery vehicle can be decorated with different functional groups which can be used to bind with the drug molecules or specific ligand molecules to enhance the drug specificity.

In the present review, we have compiled the literature reporting different PLGA-based transdermal drug delivery systems and revealed their implications over the other administration routes.

## PLGA-based transdermal drug delivery systems

### PLGA-based microneedles for transdermal drug delivery

**Fig. 1:**
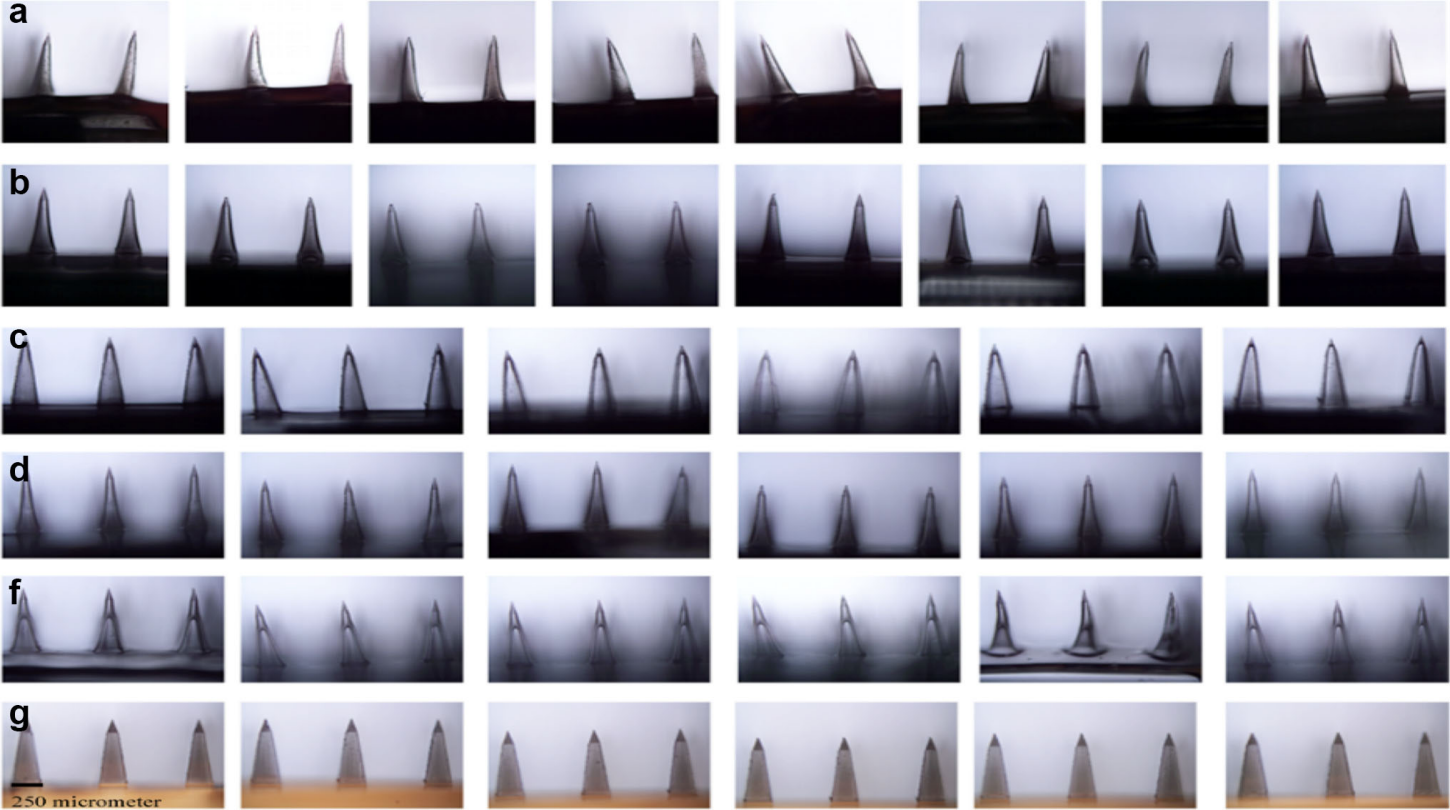
Digital photographs of sections from 10,610 dissolvable MNs fabricated from PDMS micromolds. **a** sodium alginate microneedles, **b** hydroxypropyl cellulose, **c** hydroxypropyl cellulose, **d** cross-linked swellable polyvinyl alcohol-gelatin, **e** chitosan, and **f** PLGA with permission from Demir et al. (2013)

Delivering pharmacologically active molecules into deeper layers of the skin can be achieved using microneedles (MNs), which would minimize the pain in patients, biosafety, and might be useful as self-applicable systems (Demir et al. 2013). This approach is considered as a miniaturized novel device. It possesses several needles less than 22 mm height on assembling. The use of MNs provides small hydrophilic drugs delivery, as well as the transportation of lipophilic and macromolecular therapeutics agents. MNs can deliver drugs through the stratum corneum (SC). It is of great importance to select microneedle application approach and type. Polymeric MNs have specific application in transdermal drug delivery, due to their capacity to avoid cross-contamination. In addition to that, the selected materials can be used to optimize the desired application and behavior as swelling, degradation, and dissolution. Figure 1 shows the microneedles designed using different polymers, such as sodium alginate (SA), hydroxypropyl cellulose (HPC) (M and H grades), cross-linked polyvinyl alcohol (PVA), and gelatin hydrogels, chitosan, and poly(lactic-*co*-glycolic acid) (PLGA) (Demir et al. 2013).

**Fig. 2:**
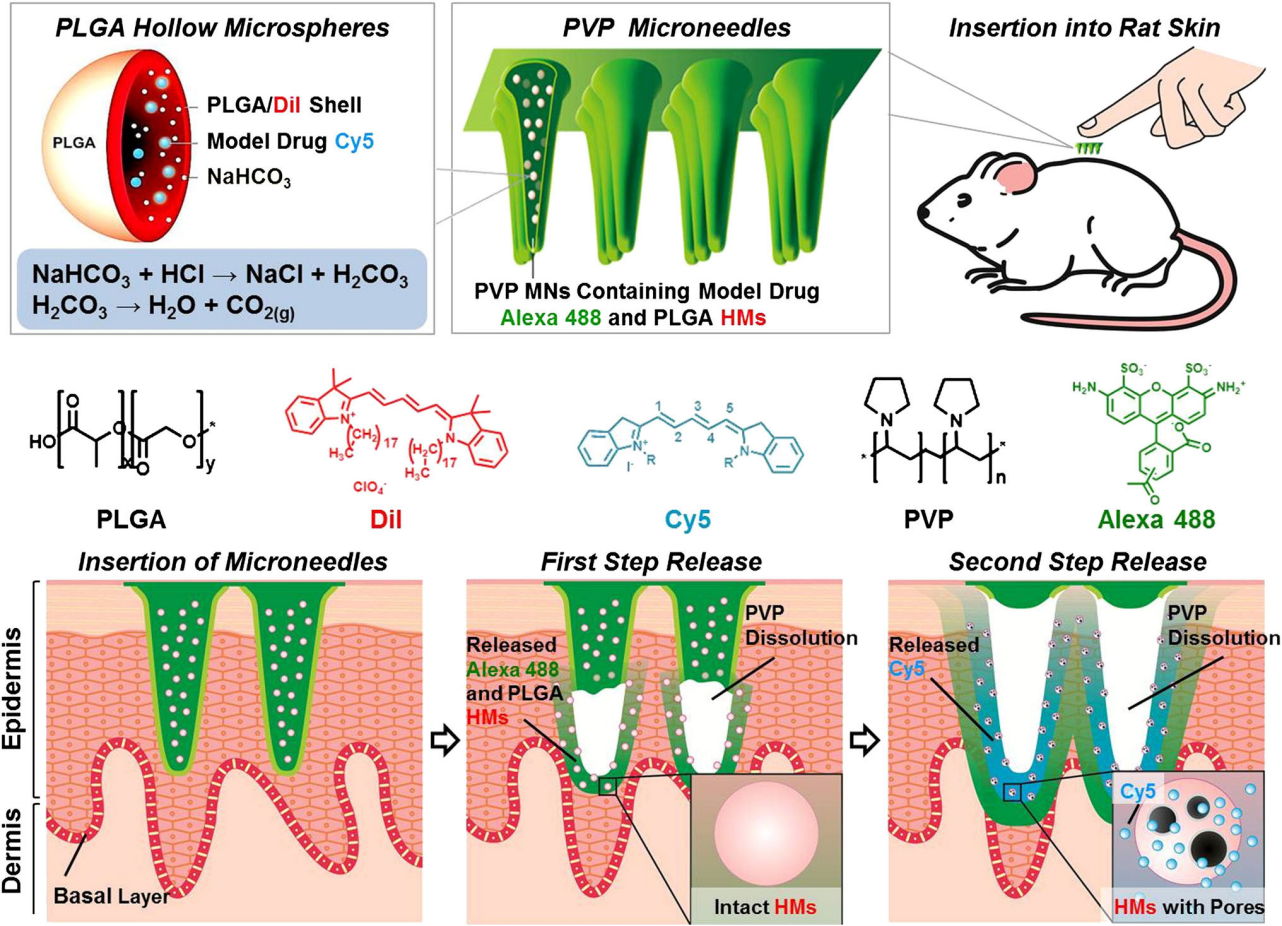
Schematic illustration of the design of PVP MN arrays containing pH-responsive PLGA HMs and their mechanism for co-delivery of two different model drugs Alexa 488 and Cy5 in sequence transdermally. After* insertion* into skin, the first step of rapid release of Alexa 488 and Dil-labeled HMs was accomplished due to quick dissolution of PVP polymers. The second-step release of Cy5 from HMs was stimulated by the acidic skin environment. PVP MNs: polyvinylpyrrolidone microneedles; PLGA HMs: poly(dl-lactic-*co*-glycolic acid) hollow microspheres With permission from Elsevier (Ke et al. 2012)

Ke et al. (2012) have reported a novel approach to deliver two model drugs transdermally. They used poly vinyl pyrrolidone (PVP) due to its strong behavior for skin insertion. PVP is approved by the US Food and Drug Administration (FDA) for many drug delivery applications. In another study, PVP has been reported to design microneedles that can be quickly dissolved in the skin, leaving behind no biohazardous sharp points and delivering the encapsulated drugs (Donnelly et al. 2011). In another study, Ke et al. have simultaneously encapsulated both Alexa 488 and Cyanine5 (Cy5) loaded PLGA microspheres together into PVP MNs. Authors have used double emulsion method to develop pH-responsive PLGA hollow microspheres (HMs); the aqueous core based on sodium bicarbonate (NaHCO_3_) and Cy5, and the shell contained Dil (1,1′-dioctadecyl-3,3,3′,3′-tetramethyl indocarbocyanine perchlorate). Subsequently, using PDMS mold, PVP MN arrays were fabricated containing PLGA HMs and Alexa 488. Healthy skin is acidic, with pH ranging from 4.2 to 5.6. However, the epidermis pH value is approximately 5.5; the skin cancer has been reported and observed to develop at the epidermis layer. This smart PLGA-based microneedle design is reported to release both encapsulated drugs in two phases and helps achieving different timescales of controlled release. As soon as they inserted the MNs into the skin, the first step that was observed was the rapid release of Dil-labeled HMs Alexa 488, related to the quick dissolution of PVP polymers. The second phase of the drug release is due to the exposure of Dil-labeled HMs Alexa 488 to the skin condition, which has acidic behavior in natural conditions. The release process happens when the HMs-w-NaHCO_3_ reacts with the acid (H^+^), generating CO_2_ bubbles, intruding through the HMs, and releasing the encapsulated Cy5 (Fig. 2).

**Fig. 3:**
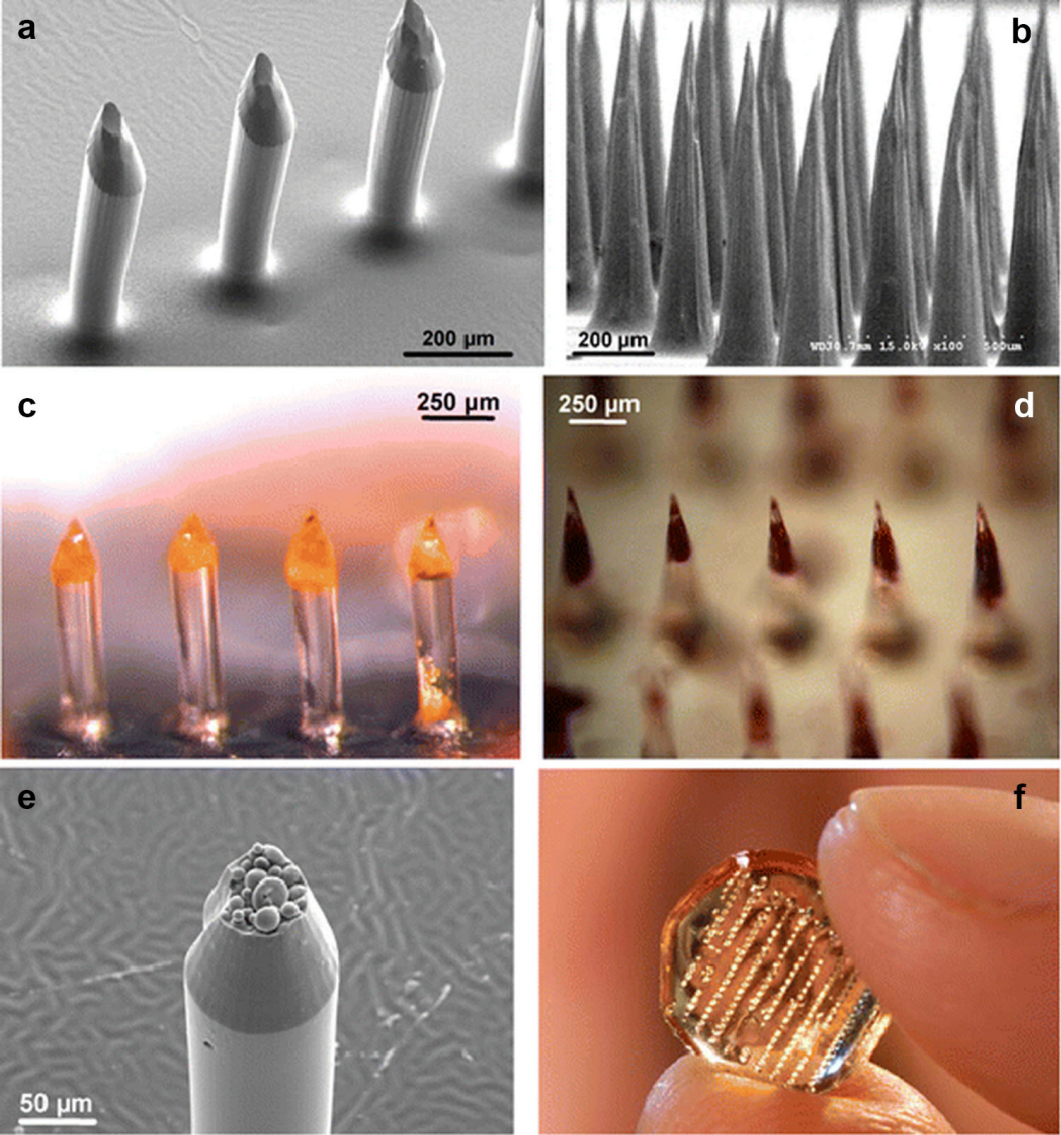
Microscopy images of microneedles. A section of an array of **a** microneedles, **b** tapered-cone microneedles, **c** bevel-tip, **d** tapered-cone microneedles made of PLGA, **e** PLGA microneedle showing microparticles, and **f** a complete PLGA microneedle arrays With permission from Springer (Park et al. 2006)

In another study (Park et al. 2006), authors have reported the development of polymericmicroneedles for developing controlled DDS, involving microelectromechanical system (MEMS) techniques which were used to make molds, fabricating microneedles based on biodegradable and biocompatible polymers (Fig. 3). The microneedles developed have the height of 600 µm, tip radius of 5 µm, and base radius of 50 µm. The entire array area is 9 × 9 mm, where the needles were positioned in a 20 × 6 arrays with center-to-center spacing between needles of 400 and 1400 µm.

## PLGA nanoparticles for transdermal drug delivery

**Fig. 4:**
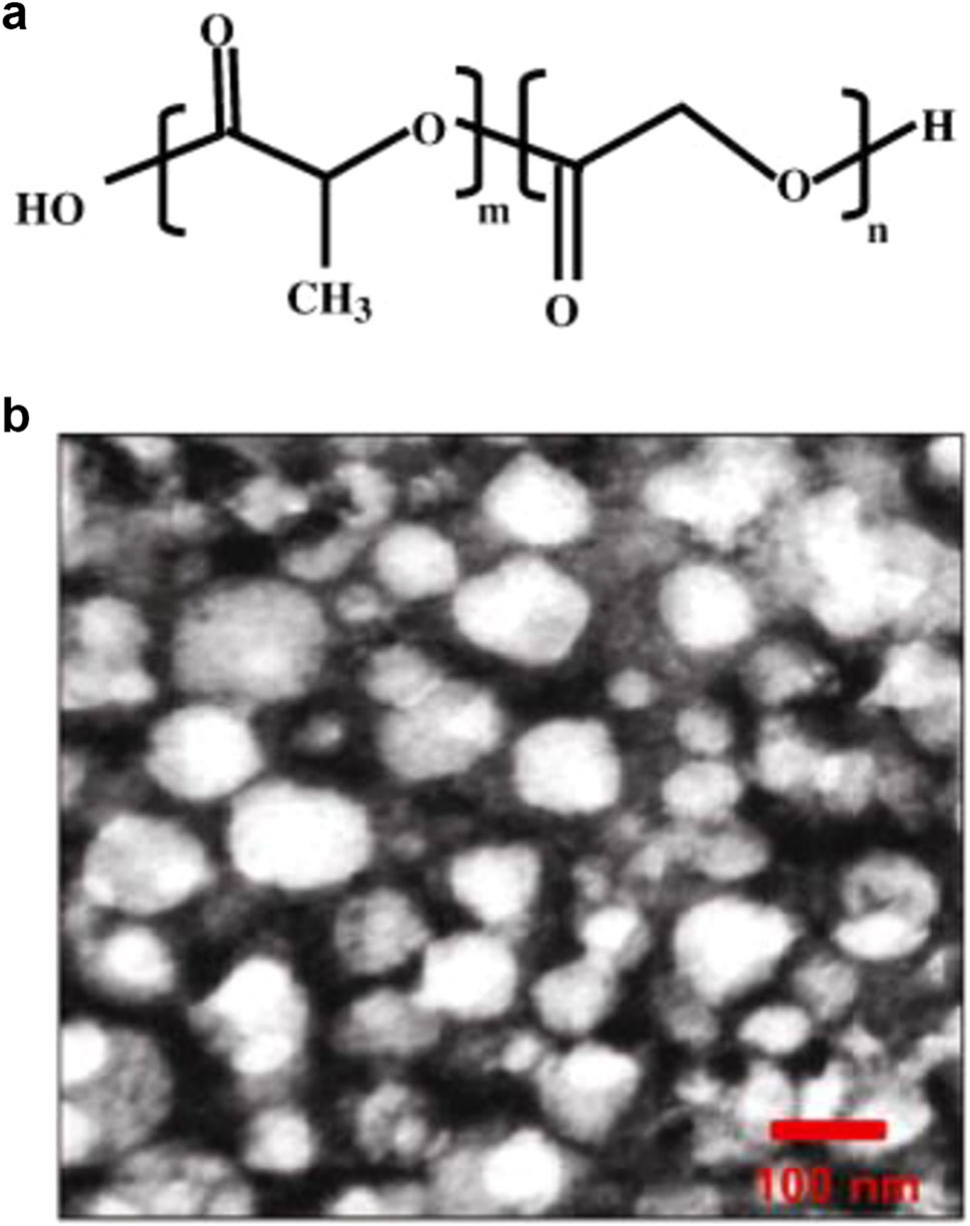
**a** Chemical structure of PLGA where, *m* represents number of units of lactic acid and *n* represents number of units of glycolic acid; **b** (TEM) of PLGA nanoparticles With permission from Elsevier (Acharya and Sahoo 2011)

A common pharmaceutical strategy is the encapsulation of active substances to modify the release properties and the transport of a drug. The nanoparticle systems are a great potential for DDS. As a consequence of the fact that, some sensitive drugs could be hidden from degradation in the particles (Stracke et al. 2006). The nanoparticles (NPs) for pharmaceutical purposes are defined as solid colloidal particles ranging in size from 10 to 400 nm. Different polymers can be used to develop nanoparticles (Dinarvand et al. 2011). Synthetic polymeric nanoparticles are the mainly used in designing DDS, since natural polymers broadly vary in their degree of purity. On the contrary, using synthetic polymers, a good porosity can be achieved and nanoparticles can be modelled (Panyam and Labhasetwar 2003). Commonly used synthetic polymers for drug delivery application, among many, include PLGA. Figure 4 presents the chemical backbone of PLGA with the TEM image showing the PLGA nanoparticles (Acharya and Sahoo 2011).

Some drug penetration evaluation methods into the skin are mostly destructive; a representative sample of defined skin layer is isolated and, therefore, extracted for chemical analysis, such as: cryo-sectioning and tape stripping method (Brain et al. 2002). As a result of such tests, it is possible to obtain some data, regarding the deep profile of drug location into the skin versus release time (Luengo et al. 2006). For the characterization of the depth, it is necessary to perform investigation using multiple samples and employing more versatile techniques to optimize and evaluate novel dermal drug delivery strategies for drugs in nanoscale size. Following the application of nanoparticles as topical vehicles, the penetration may be achieved through different routes. It can be assumed that the nanoparticles may enter once the nanocarrier is decomposed closer to the skin surface or the nanoparticulate systems are taken up without being destroyed (Kohli and Alpar 2004). Thereafter, the penetration absorption rate of the active substance may depend upon local environment, such as absorption or acidification of nanoparticles/drug complexes (Stracke et al. 2006).

**Fig. 5:**
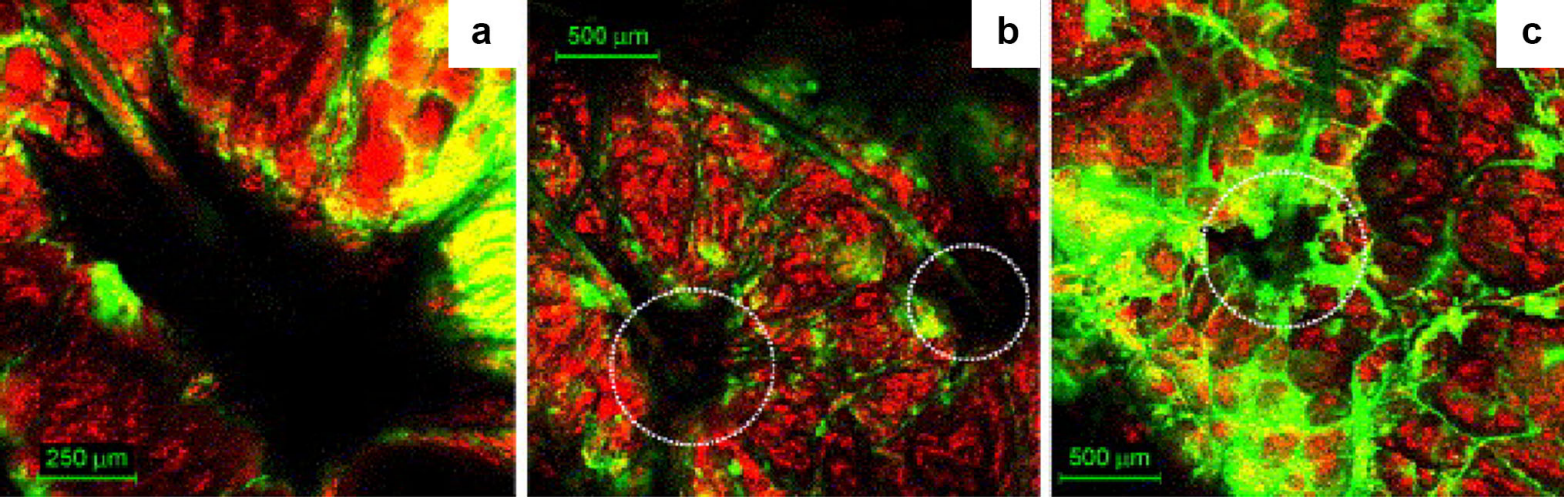
Images showing skin surface and saturated absorption of isothiocyanate followed by 30 min (**a**), 1 h (**b**), and 2 h (**c**). Possible nanoparticle route through follicular pathway (Alvarez-Romn et al. 2004) With permission from Elsevier

The particle size of PLGA is the key factor in deciding its biodistribution and therapeutic efficacy. Nanoparticles of PLGA with range size smaller than 100 nm showed higher cells uptake in the combination of polymer hydrophilicity behavior and surface charge (Bilati et al. 2005). In our skin, hair follicles occupy approximately 0.1% of the total surface area. The hair follicles play a significant role in the nanoparticles penetration, as they increase the absorption area below the skin surface. Some researchers point out that particle penetration is related to the “activity” of the follicles, likewise the sebum production and hair growth (Contreras 2007). In Fig. 5, it is possible to observe the follicular route for nanoparticles penetration, appearing as a promising approach for drug delivery.

Nanoparticles can be delivered through transdermal pathways, confirmed by the work of many researchers, and the recent development of engineered nanomaterials and the development of nanotechnology. The use of these nanomaterials is useful, since they can penetrate deeper into the protective layer of skin, delivering drugs or active agents and nutrients, such as non-synthetic peptide that instruct cells to regenerate, as some of these nanoparticles have antioxidant properties (Xiao et al. 2005). The gamut of nanoparticles used in products delivered through transdermal pathways is assumed to have health effects which are not yet known, though some of these nanoparticles have been considered safe in the past. Nonetheless, there are now some nano-toxicological concerns; as an example, we can cite silver, which has widely been used due to its antimicrobial activity. Nowadays, it is found that silver at nanoparticle scale might provoke adverse toxicity effects to animals and humans (Soto et al. 2005). Inhalation of the silver nanoparticle can provoke acute problems to the circulatory systems, heart, and kidneys (Takenaka et al. 2001). In this case, the use of nanoparticle-sized materials instead of helping the patients to fight their disease can lead to several unpleasant complications that may cause severe infection or even death. The small nanoparticle size can influence some important cellular regulatory process, such as proliferation, metabolism, and death. The dysfunction of these essential cellular processes can be associated with many diseases as well as a neurodegenerative disease or cancer when the disease causes part of premature cell death or uncontrolled cell proliferation, respectively (Antonini et al. 2006).

Nanoparticles can enter cells like nano-organisms, as viruses for example. By doing so, once these nanoparticles are absorbed, they can interact with subcellular structures and mechanisms. The nanoparticle size, chemistry, and shape are directly related to the cellular uptake, ability to catalyze oxidative products and subcellular localization (Xia et al. 2006). Molecules with a diameter of 0.7 nm can penetrate cells via different mechanisms, probably through pores in the cell membrane or ion channels (Porter et al. 2006). This type of free movement and uptake of the cell might have harmful consequences, once it is easily accessible to cytoplasm organelles and proteins. Depending on the localization of the nanoparticles inside the cells, they can damage the DNA or organelles, or ultimately cause cell death. The nanoparticles can be found in different regions inside the cells, upon non-phagocytic uptake, such as the outer membrane, mitochondria (Li et al. 2003), cytoplasm, and lipid vesicles (Garcia-Garcia et al. 2005), within the nucleus (Xia et al. 2006) or along the nuclear membrane.

## PLGA nanofibers for transdermal drug delivery

Medication of drugs plays important role in the medical field. However, most drugs are absorbed only when their concentration in the blood is above their minimal effective level. Each drug has its own half-life and it is not possible to maintain the concentration for a long time. By increasing the drug dosage, subsequently, the patients are under a higher toxicity risk, which is not convenient for the patient (Meng et al. 2011). Electrospinning technique has received considerable interest for nanofiber engineering to produce new systems for drugs delivery through transdermal pathways. Using this method, it is possible to fabricate porous nanofibrous materials with some specific characteristics like three-dimensional morphologies, high porosity, large surface area, and volume ratio. The use of this technique allows manufacturing nanofibers from natural or synthetic polymers. Electrospun nanofibers can be used as template for the production of drug loaded polymers. These biodegradable polymers as drug carriers can act as an adjuvant, protecting the drug from corrosion of enzymes and gastric acid, preserving the drug activity (Meng et al. 2011).

Ajalloueian and co-workers (2014) stated that: lactide and glycolide are the most used synthetic polymers for electrospinning technique. These synthetic polymers have excellent mechanical properties and are biodegradable. Its hydrophobic structure results in a reduced cell-scaffold attachment interaction due to the lower surface energy. The product degradation is related to its acidity and negative surface charge.

Several factors might interfere with the electrospinning process, such as emulsion concentration, feed rate, applied voltage, and tip to collector distance. By reducing the polymer concentration in the emulsion, the average diameter of the electrospun nanofiber is decreased, which is related to the lower emulsion viscosity (Meng et al. 2010). Higher concentration of PLGA produces an emulsion which is very viscous for electrospinning.

It has been reported (Ajalloueian et al. 2014) that, when using high voltage of 16 kV, it is possible to achieve more uniform nanofiber; by decreasing the voltage to 12 kV, the nanofibers are less consistent; and at the voltage of 8 kV, it is possible to observe the formation of some beads along the electrospun nanofibers. The higher the voltage applied to the process of electrospinning, the better properties are obtained as more uniform, beads-free, and finer nanofiber formation. The distance between the Taylor cone and the collector in the range of 8–15 cm does not show any significant effect on the diameter of electrospun.

**Fig. 6:**
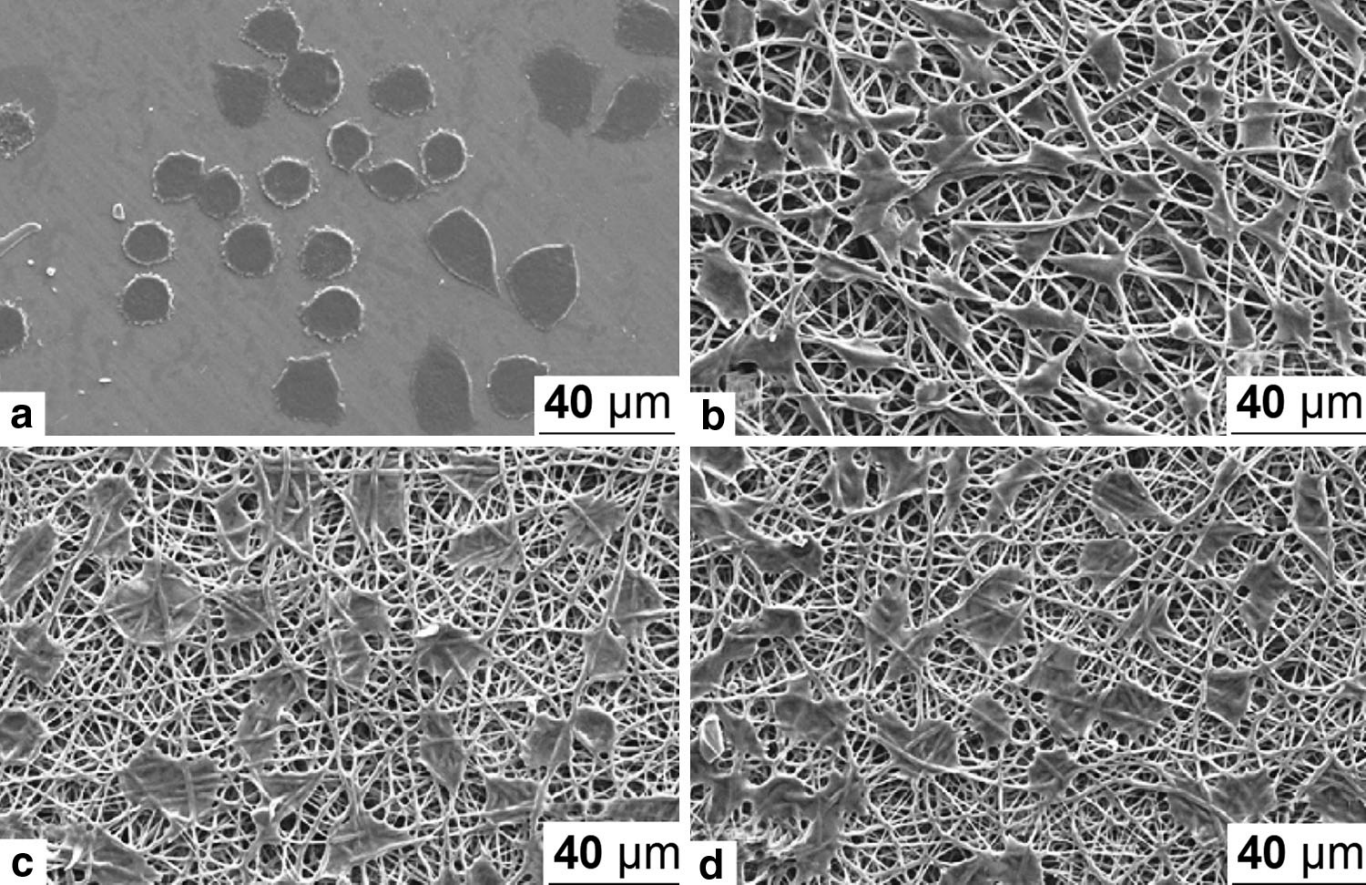
Human dermal fibroblast growth **a** tissue culture plate (TCP), **b** PLGAmats, MWCNTs/PLGA nanofibers with 3 wt% (**c**) and 5% (**d**) of multi-walled carbon nanotubes relative to PLGA With permission from Springer (Qi et al. 2016)

**Fig. 7:**
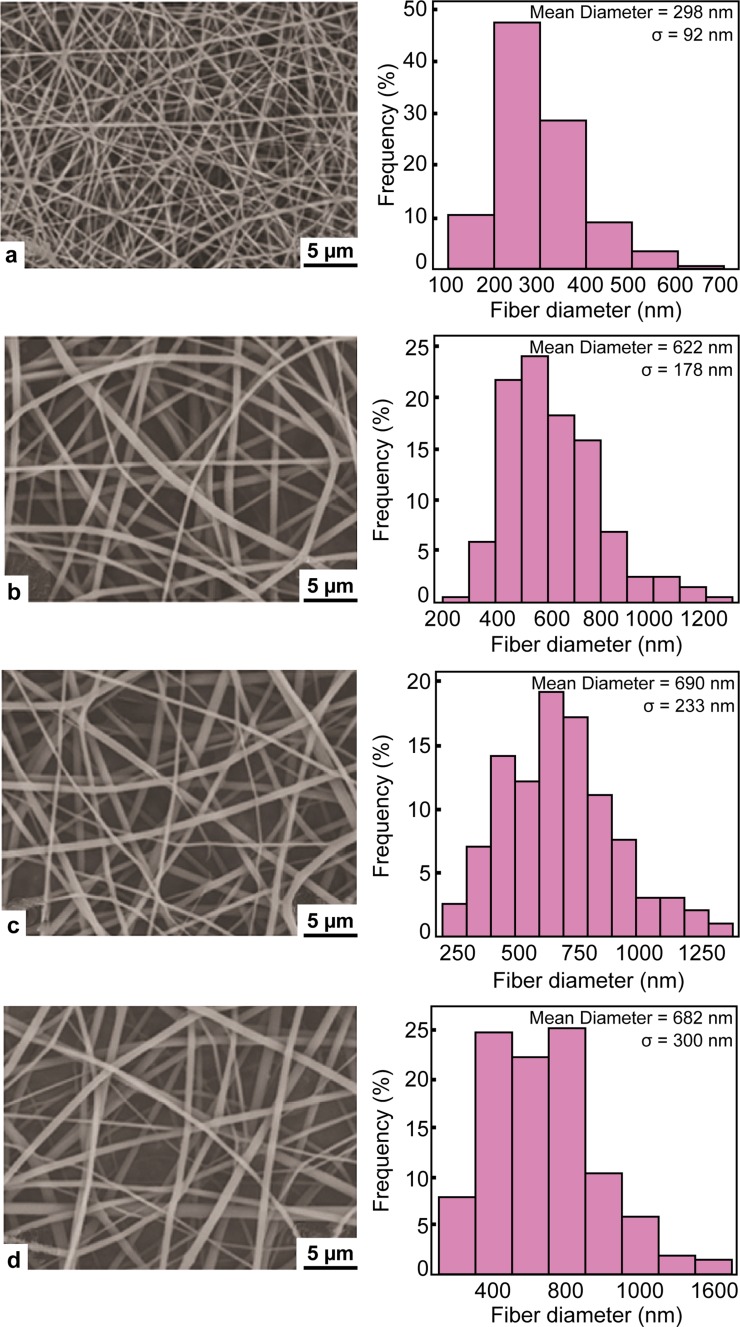
SEM images and corresponding fiber diameter distribution histograms of the electrospun mats. **a** Doxorubicin blended with PLGA 1 wt % of Doxorubicin relative to PLGA, **b**, **c, d** blended mats of doxorubicin/multi-walled carbon nanotubes and PLGA containing, respectively, 1, 2, and 3 wt% of doxorubicin relative to PLGA With permission from Springer (Qi et al. 2016)

In another study (Qi et al. 2016), there was a report on the development of doxorubicin (Dox), loaded with PLGA and multi-walled carbon nanotubes (MWCNTs) to encapsulate a model anticancer drug for the development of electrospun mats. The biocompatibility, cell proliferation, and greater adhesion of dermal fibroblasts on the surface of the mats can be observed in Fig. 6. SEM micrographs of this electrospun mats are shown in Fig. 7. The authors have reported that after loading doxorubicin into PLGA solution, the Dox/PLGA bleb fibers were much smaller than pure PLGA fibers. It is known that the electrospinning solution property can significantly be affected by the addition of cationic or anionic species. The introduction of doxorubicin, cationic drug, may cause an increase in the surface density of the spinning jet, therefore, resulting in a smaller fiber diameter. The authors have reported that the use of these biocompatible electrospun mats might have a potential application in tissue engineering and local post-operative chemotherapy.

The extracellular matrix (CCM) is the natural scaffold for most of tissues, whose morphology and structure contribute greatly to the function and properties of each organ. It can be an adjuvant for the elasticity of the skin (Bottaro et al. 2002). To mimic the natural function of the organs, many researchers are exploring the possibility of using and developing new copolymers biochemical/biopolymers, incorporating growth factor, biological agents, and some other key cell regulatory molecules (Luu et al. 2003).

## Nanostructure evaluation techniques

### Scanning electron microscopy

Scanning electron microscopy (SEM) technique can be used to visualize and analyze the surface morphology of the nanostructures, including nanoparticles, nanofibers, microspheres, etc. It employs finely focused electron beam to image the surface of the specimen. This technique was explained by Jacob (1952) as per which the instrument consists of an electron gun, which produces an electron beam, focused on the specimen surface. The computer memory is measured and stored the intensities of various signals created by the interaction between the sample scanned area and the size displayed images.

### Energy-dispersive X-ray spectroscopy

This technique can be used to do the chemical characterization or/and elemental analysis of the sample, and it determines the quantitative elemental composition of the sample. This characterization is based on the fact that each chemical element has its unique atomic structure which allows to set-up individual peaks on its X-ray emission spectrum. A high-energy beam of X-rays or beam of charged particles like protons or electrons is focused into the sample. Before the high-energy beam incident on the sample, the sample is found in the unexcited state where the atoms are in discrete energy level. The exposed beam provokes an excitement of the electrons found in the inner shell, ejecting it from the shell, consequently, creating electron hole. This phase is followed by the removal of an electron from an outer higher energy shell filling the hole, as a result of this ejection of the electron from the shell, an X-ray is released, and it is the difference in energy between the high-energy shell and the lower energy shell. The energy of X-rays emitted from the sample is measured by the energy-dispersive spectrometer (Hafner 2006).

### X-ray diffraction

X-ray diffraction (XRD) relies on the dual wave/particle nature of X-rays to obtain information about the structure of crystalline materials. This technique is used for evaluating crystallinity of the polymers. In 2008, Petkov (2008) stated that the atomic-scale structure of materials is determined by X-ray diffraction technique. By doing so, it is possible to analyze the distance between atoms in condensed matter, which is comparable to the wavelengths in X-ray range. When a material, such as crystal, is irradiated with X-rays, it exhibits Bragg diffraction peaks, which is the diffraction pattern showing numerous sharp spots. The arrangement of the atoms in the crystalline materials and its 3D atomic position can be determined by analyzing and measuring the intensities and the position of Bragg diffraction peaks.

### Transmission electron microscopy

While using transmission electron microscopy, the important components are vacuum system, an electron optical column, the necessary electronics (the high voltage generator for the electron source and the lens to focus and deflect beams). This technique is suitable for observing animal and plants cells at high magnifications, to obtain cell information. The specimens must be thin, for achieving the transition of the electron beams, typically 0.5 µm or less. Using high accelerating voltages, higher energy of electrons is obtained, which can penetrate the thicker samples. Vacuum is used to perform this technique, which allows the entire electron path from the gun into the camera. Otherwise, the electron beams would collide with air molecules and absorbed or scattered (Jacob 1952).

## Conclusions

In conclusion, with the growing advancements in the field of nanotechnology, new paradigms have developed in novel approaches and strategies to design efficient nanomedicines targeting different diseases. Efficient drug delivery system (DDS) pertains to the chemically stable biodegradable delivery vehicles that can release drug at the targeted tissue or area, minimize the therapeutic side-effects, and also conserve sustained release for a longer duration. PLGA is found to be a promising synthetic polymer for different biomedical applications, including their implications in designing competent drug delivery systems. Using PLGA, it is possible to encapsulate variety of drugs like hydrophilic or hydrophobic with size ranging from small molecules to macromolecules to deliver them at various targeted locations using different administration routes, including intravenous drug delivery, pulmonary drug delivery, transdermal drug delivery, etc. Further to enhance the loading efficiency and specificity of the drug delivery carrier, PLGA surface properties can be tuned using different coating protocols and functionalization strategies. PLGA is also reported as efficiently designed in DDS for transdermal drug delivery in the form of microneedle arrays, nanoparticles, and nanofiber mats. SEM, TEM, EDX, and XRD are some of the morphological and structural analysis techniques to study PLGA micro- and nanostructure-based DDS.
